# Pharmacological manipulation of arachidonic acid-epoxygenase results in divergent effects on renal damage

**DOI:** 10.3389/fphar.2014.00187

**Published:** 2014-08-15

**Authors:** Jing Li, Charles T. Stier, Praveen N. Chander, Vijay L. Manthati, John R. Falck, Mairéad A. Carroll

**Affiliations:** ^1^Department of Pharmacology, New York Medical CollegeValhalla, NY, USA; ^2^Department of Pathology, New York Medical CollegeValhalla, NY, USA; ^3^Department of Biochemistry, University of Texas Southwestern Medical CenterDallas, TX, USA

**Keywords:** epoxyeicosatrienoic acids, SHRSP, high-salt intake, renal damage, proteinuria, clofibrate, epoxygenase inhibition

## Abstract

Kidney damage is markedly accelerated by high-salt (HS) intake in stroke-prone spontaneously hypertensive rats (SHRSP). Epoxyeicosatrienoic acids (EETs) are epoxygenase products of arachidonic acid which possess vasodepressor, natriuretic, and anti-inflammatory activities. We examined whether up-regulation (clofibrate) or inhibition [*N*-methylsulfonyl-6-(2-propargyloxyphenyl)hexanamide (MS-PPOH)] of epoxygenase would alter systolic blood pressure (SBP) and/or renal pathology in SHRSP on HS intake (1% NaCl drinking solution). Three weeks of treatment with clofibrate induced renal cortical protein expression of CYP2C23 and increased urinary excretion of EETs compared with vehicle-treated SHRSP. SBP and urinary protein excretion (UPE) were significantly lowered with clofibrate treatment. Kidneys from vehicle-treated SHRSP, which were on HS intake for 3 weeks, demonstrated focal lesions of vascular fibrinoid degeneration, which were markedly attenuated with clofibrate treatment. In contrast, 2 weeks of treatment with the selective epoxygenase inhibitor, MS-PPOH, increased UPE without significantly altering neither urinary EET levels nor SBP. Kidneys from vehicle-treated SHRSP, which were on HS intake for 11 days, demonstrated occasional mild damage whereas kidneys from MS-PPOH-treated rats exhibited widespread malignant nephrosclerosis. These results suggest that pharmacological manipulation of epoxygenase results in divergent effects on renal damage and that interventions to increase EET levels may provide therapeutic strategies for treating salt-sensitive hypertension and renal damage.

## INTRODUCTION

Salt-sensitivity is an important characteristic of a subgroup of humans with essential hypertension (Katori and Majima, 2008). In particular, high dietary salt increases the susceptibility of hypertensive patients to renal damage (Cowley and Roman, 1996). The stroke-prone substrain (A_3_N) of the spontaneously hypertensive rat (SHR), or stroke-prone SHR (SHRSP), is a well-established model of genetic hypertension in which end-organ damage is highly salt-sensitive. Excess dietary salt dramatically increases the onset of stroke, myocardial infarction, and renal damage while only moderately elevating blood pressure (BP) further in SHRSP (Zuckerman et al., 1997). Previous studies in SHRSP using agents that interfere with the renin–angiotensin–aldosterone system have shown a dissociation between BP lowering and protection against salt-sensitive end-organ damage (Stier et al., 1991; Rocha et al., 1998) and have specifically implicated aldosterone as a major factor in the etiology of salt-sensitive kidney damage (Chander et al., 2003). Consistent with a pivotal pathophysiological role of aldosterone in the saline-drinking SHRSP and the ability of aldosterone to stimulate the epithelial sodium channel (ENaC), Sepehrdad et al. (2003, 2004) found that amiloride and other agents that inhibit ENaC function offer protective effects against the development of proteinuria and renal microvascular damage. Interestingly, epoxyeicosatrienoic acids (EETs), cytochrome P450 (CYP) epoxygenase metabolites of arachidonic acid, have been shown to directly inhibit ENaC activity (Wei et al., 2004). EETs not only block the action of aldosterone at the level of the distal nephron, but they are also involved in the long-term regulation of BP and in the functional response of the kidney to high-salt (HS) diet (Liclican et al., 2008, 2009). Studies in experimental animal models also provide substantial evidence for EETs in the regulation in inflammation, fibrosis, and platelet aggregation (Node et al., 1999; Krotz et al., 2004). These properties of EETs enable them to serve in a variety of settings to protect and sustain both renal and systemic circulatory function. Therefore, we reasoned that interventions directed at induction or inhibition of epoxygenases responsible for the production of EETs might have a major impact on the pathologic renal changes that occur in saline-drinking SHRSP.

There are two pharmacological approaches that have been used to chronically elevate endogenous levels of EETs in order to evaluate their renal and vascular protective actions *in vivo*. One approach is to inhibit the conversion of EETs to their less active metabolites, dihydroxyeicosatrienoic acids (DHETs), by soluble epoxide hydrolase (sEH; Zeldin et al., 1995). We have shown that *in vivo* treatment with a sEH inhibitor, 2-(3-adamantan-1-yl-ureido)-dodecanoic acid (AUDA), prevented the early salt-sensitive components of hypertension and kidney damage in saline-drinking SHRSP (Li et al., 2008). Another approach is to increase levels of EETs by inducing epoxygenases with fibric acid derivatives such as clofibrate, fenofibrate, and bezafibrate (Muller et al., 2004; Zhao et al., 2006). Fenofibrate has been shown to strongly induce renal protein expression of CYP2C23, a major CYP epoxygenase in the rat kidney, and increase renal epoxygenase activity (Muller et al., 2004). On the other hand, epoxygenases can be inhibited with *N*-methylsulfonyl-6-(2-propargyloxyphenyl)hexanamide (MS-PPOH), which has been identified as a potent and selective inhibitor of CYP-catalyzed arachidonate epoxidation both *in vitro* (Brand-Schieber et al., 2000) and *in vivo* (Liclican et al., 2009). We have previously reported that *in vivo* MS-PPOH treatment significantly reduced renal levels of EETs and rendered Dahl salt-resistant rats hypertensive (Liclican et al., 2009). In the present study, we hypothesized that epoxygenase stimulation would reduce BP and protect against renal damage in saline-drinking SHRSP, whereas inhibition of epoxygenase activity would increase BP and exacerbate renal damage in these animals.

## MATERIALS AND METHODS

### ANIMALS

Six-week-old male SHRSP, bred from NIH stock derived originally from the SHRSP/A_3_N substrain, were obtained from Charles River Laboratories. Rats were given standard rodent diet (Purina Lab Chow # 5001, 0.38% Na^+^ and 1.23% K^+^; Stier et al., 1989) and allowed tap water *ad libitum*. Animals were housed in a temperature-controlled room with a 12-h light/dark cycle and were used in accordance with NIH guidelines. The New York Medical College Institutional Animal Care and Use Committee approved all experimental protocols.

### CLOFIBRATE TREATMENT OF SHRSP

SHRSP were maintained on stroke-prone rodent diet (0.38% Na and 0.71% K, Zeigler Brothers, Gardners, PA, USA; Stier et al., 1989) and 1% NaCl drinking solution starting at approximately 7 weeks of age. Clofibrate (200 mg/kg/day, *n* = 7; Sigma–Aldrich, St. Louis, MO, USA) or vehicle (0.5% methylcellulose, *n* = 5) administered once daily by gavage, was started 3 days prior to giving SHRSP 1% NaCl drinking solution. Systolic BP (SBP) was measured weekly using tail-cuff plethysmography (CODA 2 non-invasive BP apparatus, Kent Scientific, Torrington, CT, USA). After 3 weeks of HS intake, animals were housed in metabolic cages and urine was collected for measurement of eicosanoids by Liquid Chromatography/Mass Spectrometry/Mass Spectrometry (LC/MS/MS) analysis and urinary protein excretion (UPE) by the sulfosalicylic acid turbidity method (Stier et al., 1989). Rats were then anesthetized with sodium pentobarbital (65 mg/kg, i.p.) and kidneys were excised and sections of cortex were snap frozen in liquid N_2_ for Western immunoblot analysis and the remaining kidney was placed in formalin for histological evaluation.

### MS-PPOH TREATMENT OF SHRSP

The right jugular vein of 12 SHRSP was cannulated at 6 weeks of age as previously described (Liclican et al., 2009) and the animals were allowed 1 week for recovery. Animals were switched to stroke-prone rodent diet and treated with MS-PPOH (20 mg/kg/day, synthesized by Dr. John R. Falck, Texas Southwestern Medical Center, TX, USA; *n* = 7) or vehicle (45% hydroxypropyl β-cyclodextrin, 1.5 ml/kg/day; *n* = 5) as bolus injections into the jugular vein catheter twice per day, starting at 7 weeks of age. SHRSP were given 1% NaCl to drink 3 days after the treatment with MS-PPOH was started. SBP was measured and urine was collected weekly after MS-PPOH treatment. UPE was measured weekly and the end point of the study was based on the occurrence of proteinuria, defined as a UPE of at least 20 mg/day. At that time one animal from each group was anesthetized and tissue samples were obtained as described in the above section for clofibrate.

### ANALYSIS OF URINARY EICOSANOIDS

An internal standard mixture containing 500 pg of d_8_-11, 12-EET (Biomol, Plymouth Meeting, PA, USA), d_11_-11, 12-DHET, and d_6_-20-hydroxyeicosatetraenoic acid (20-HETE; Cayman Chemical, Ann Arbor, MI, USA) was added to 2 ml of urine and lipids were extracted with Bond Elut-Certify II columns (Varian, Lake Forest, CA, USA). Briefly, each sample was diluted with 2 ml of 0.1 M sodium acetate solution (pH 7.0) containing 5% methanol, and the pH was adjusted to 6.0 with acetic acid. Columns were preconditioned with 2 ml of methanol, followed by 2 ml of 0.1 M sodium acetate solution (pH 7.0) containing 5% methanol before application of the urine samples. The columns were washed with 2 ml of methanol–water (1:1 by volume), and urinary eicosanoids were eluted with 2 ml of hexane: ethyl acetate (75:25 by volume) containing 1% acetic acid. The organic extracts were evaporated to dryness under N_2_ and reconstituted in 100 μL of methanol (Rivera et al., 2004). Extracted samples were analyzed by a quadruple linear ion trap LC/MS/MS system (Q-Trap 3200) equipped with a Turbo V ion source operated in negative electrospray mode as previously described (Liclican et al., 2009). Data were analyzed with Analyst 4.02 software.

### WESTERN IMMUNOBLOT ANALYSIS

Western immunoblotting was performed using an infrared fluorescence system (Odyssey; LI-COR Biosciences, Lincoln, NE, USA) as previously described (Liclican et al., 2008). Briefly, proteins were separated on a 10% SDS-PAGE gel and transferred to a polyvinylidene difluoride membrane. Membranes were blocked at room temperature for 1 h and incubated overnight at 4∘C with the following primary antibodies: CYP2C23 (a generous gift from Dr. J. Capdevila, Vanderbilt University, TN, USA), CYP2C11 (Oxford Biomedical Research, Oxford, MI, USA), and CYP4A (Daiichi Chemical Co, Japan). The membranes were then incubated at room temperature for 1 h with corresponding IRDye secondary antibodies (LI-COR). Some membranes were stripped of bound antibodies and reprobed with a β-actin antibody. The intensity (densitometric units) ratio of target protein to β-actin on the same membrane was calculated and used for quantitative comparisons.

### HISTOLOGICAL EVALUATION

Sections of kidney were fixed in 10% neutral-buffered formalin and embedded in paraffin blocks. The sections were cut at a thickness of 2–4 μm and stained with hematoxylin and eosin or periodic acid-Schiff reagent (PAS) for examination by light microscopy as previously described (Zuckerman et al., 1997; Chander et al., 2003; Li et al., 2008). Histologic sections were examined by a renal pathologist (Praveen N. Chander) without the prior knowledge of the treatment. For renal vascular damage, the prevalence of pathologic lesions was quantified by counting the number of vascular profiles exhibiting fibrinoid degeneration/necrosis and proliferative vasculopathic lesions. Fibrinoid degeneration of vessels was defined as the absence of myocytic nuclei in conjunction with hypereosinophilia in an area of the vessel wall and/or accumulation of brightly, PAS positive globular material in the vessel wall. The data were expressed as the total number of vessels affected per field of 200 glomeruli (Li et al., 2008). For glomerular damage, glomerular necrotizing and proliferative lesions were counted and expressed also as per field of 200 glomeruli. Tubular protein casts were counted and expressed as number of tubules presenting casts per field of 200 glomeruli.

### STATISTICAL ANALYSES

BP data were analyzed using a two-way ANOVA followed by Bonferroni *post hoc* test. Fisher’s exact test was used to determine treatment effects on renal pathology. A Student’s *t*-test was used to evaluate the protein expression. All other data were log-transformed to stabilize the variance before analyzing by Student’s *t*-test. These data are displayed using the actual values. Differences were considered statistically significant at *P*< 0.05. Data are expressed as means ± SEM.

## RESULTS

### EFFECT OF CLOFIBRATE TREATMENT ON SBP

**FIGURE 1:**
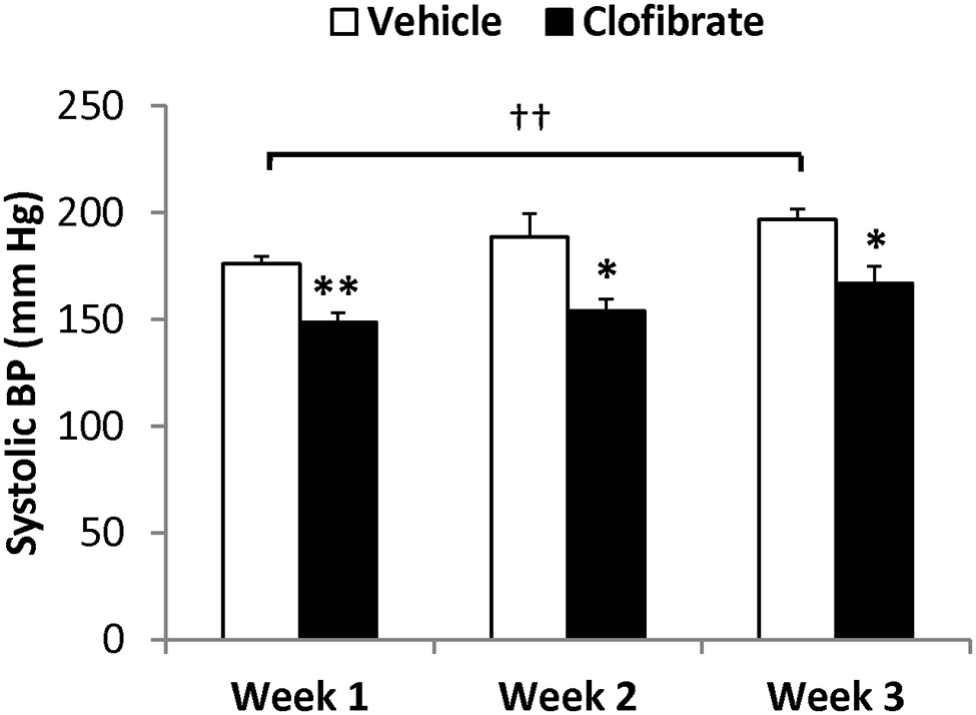
**Systolic BP of saline-drinking SHRSP treated with vehicle (0.5% methylcellulose, *n* = 5) or clofibrate (200 mg/kg/day, p.o., *n* = 7) after 1, 2, and 3 weeks of the treatment.** Data are expressed as means ± SEM; *P < 0.05, **P < 0.01 vs. vehicle; ^††^*P* < 0.01.

**Figure 1** shows results for SBP. In vehicle-treated SHRSP, SBP progressively increased from 176 ± 4 mmHg at 1 week to 197 ± 5 mmHg at 3 weeks of the study (*P* < 0.01). SBP was significantly lower in clofibrate-treated SHRSP at each week of the study (149 ± 4 mmHg at 1 week, *P* < 0.01 vs. vehicle; 167 ± 8 mmHg at 3 weeks, *P* < 0.05 vs. vehicle).

### EFFECT OF CLOFIBRATE TREATMENT ON UPE AND RENAL DAMAGE

**FIGURE 2:**
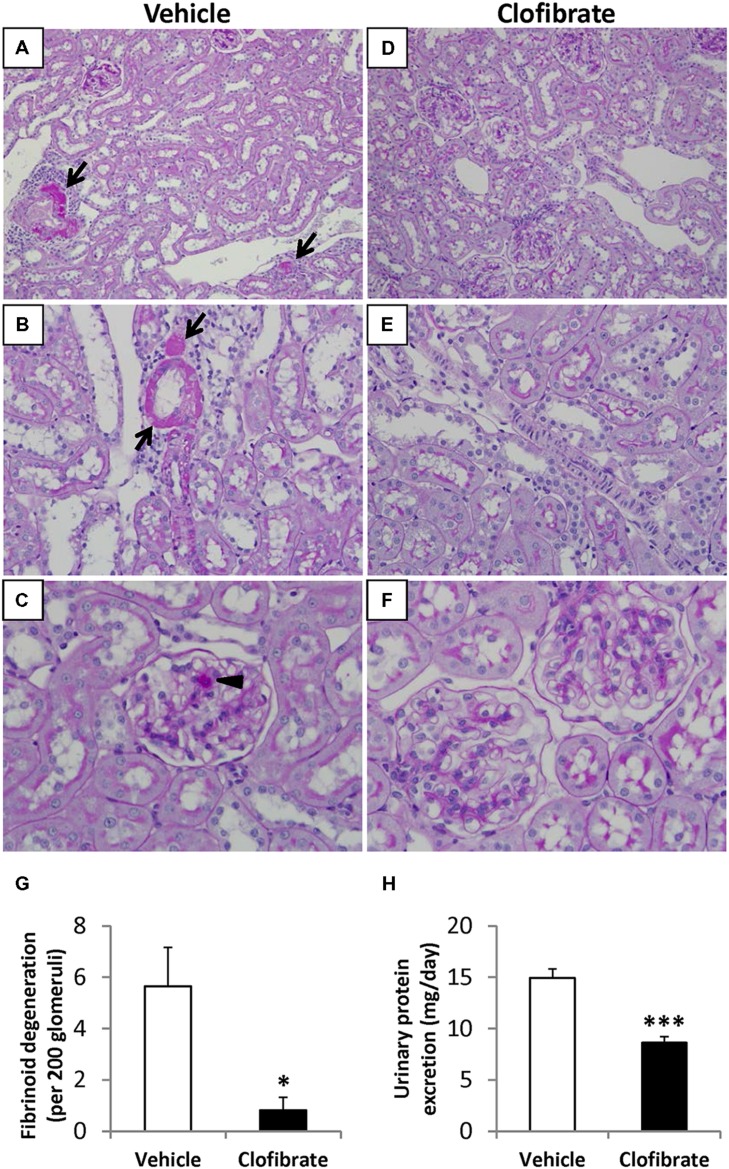
**(A–F)** Representative PAS-stained photomicrographs of renal cortical sections from three of the vehicle-treated (0.5% methylcellulose, *n* = 5, **A–C**) and three of the clofibrate-treated (200 mg/kg/day, p.o., *n* = 7, D**–**F) saline-drinking SHRSP at 3 weeks of the study. Renal sections from vehicle-treated SHRSP demonstrated focally prominent fibrinoid degeneration in vascular walls (arrows). The surrounding interstitium displays mononuclear leukocyte infiltration. Arrow head indicates prominent intracytoplasmic hyaline droplets in a glomerulus from a vehicle-treated SHRSP. Kidney sections from clofibrate-treated animals exhibited very scattered and mild vascular lesions. Magnification: ×20 **(A,D)**, ×40 **(B,E)**, or ×60 **(C,F)**. **(G)** Quantification of renal microvessels exhibiting fibrinoid degeneration per field of 200 glomeruli. **(H)** Urinary protein excretion of SHRSP treated with vehicle or clofibrate at 3 weeks of the study. Data are expressed as means ± SEM; *P < 0.05, ***P < 0.001 vs. vehicle.

Pre-terminal UPE was significantly lower in SHRSP treated with clofibrate compared with vehicle-treated SHRSP (*P* < 0.001, **Figure 2H**). Representative PAS-stained photomicrographs of renal cortical sections are provided in **Figures 2A–F**. In vehicle-treated SHRSP, three weeks of HS intake produced moderate renal pathologic lesions, consisting primarily of vascular fibrinoid degeneration. The number of vessels exhibiting fibrinoid degeneration was significantly lower in clofibrate-treated SHRSP (*P* < 0.05, **Figure 2G**). Leukocyte infiltration was present in the interstitium surrounding vessels that exhibited fibrinoid degeneration in vehicle-treated SHRSP and was abrogated in clofibrate-treated SHRSP. Tubular protein casts were present in all (5/5) kidneys from vehicle-treated SHRSP whereas only one out of seven (1/7) kidneys from clofibrate-treated SHRSP presented focal casts (*P* < 0.05). Consistent with reduced UPE and tubular protein casts, kidney sections from clofibrate-treated SHRSP showed less vascular hypertrophy and hyaline droplets in podocytes (arrowhead, **Figure 2C**) compared with vehicle-treated SHRSP.

### EFFECT OF CLOFIBRATE TREATMENT ON RENAL PROTEIN EXPRESSION OF EPOXYGENASE AND ω-HYDROXYLASE

**FIGURE 3:**
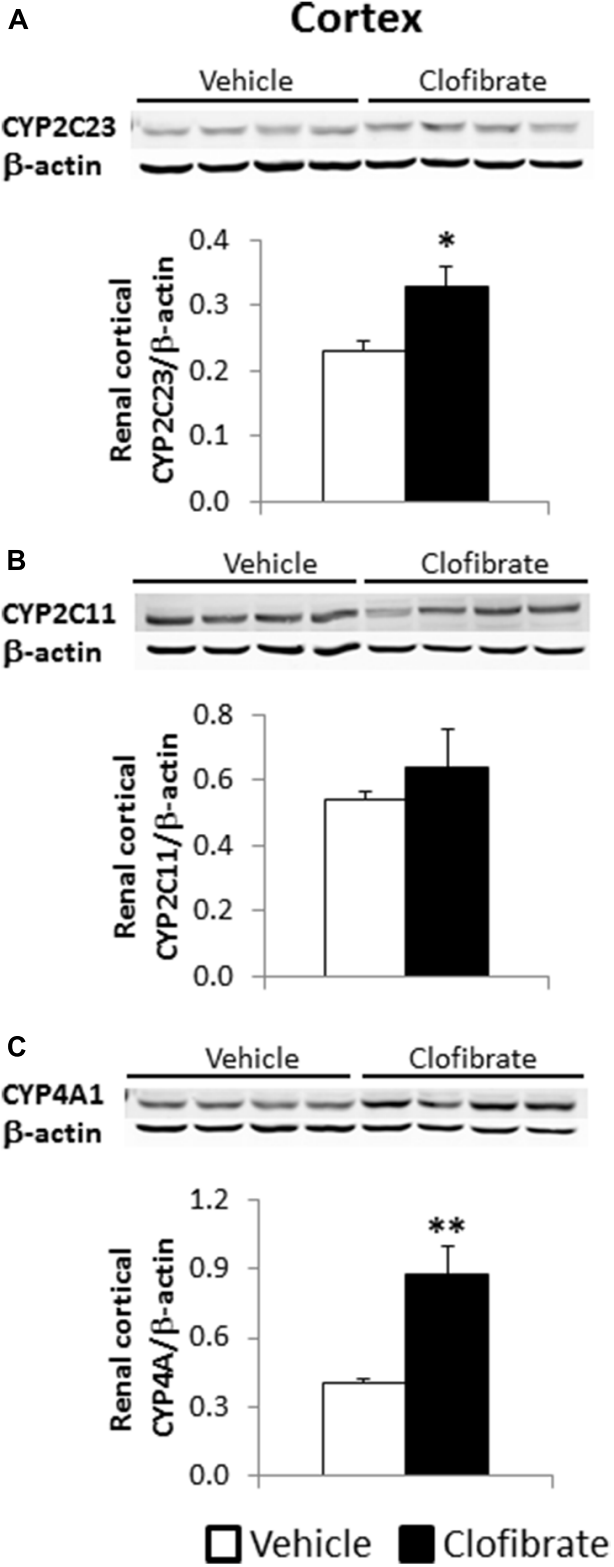
**Western blots and semi-quantitative summary of renal cortical protein expression of CYP2C23 (**A**, 48 kDa), CYP2C11 (**B**, 55 kDa) and CYP4A (**C**, 50 kDa) in saline-drinking SHRSP treated with vehicle (0.05% methylcellulose) or clofibrate (200 mg/kg/day, p.o.) for 3 weeks.** Renal cortical protein expression of CYP2C23 and CYP4A was significantly higher in clofibrate-treated SHRSP. Data are expressed as means ± SEM; *n* = 4. *P < 0.05, **P < 0.01 vs. vehicle.

Renal cortical protein expression of CYP2C23 was higher in clofibrate- compared with vehicle-treated SHRSP (*P* < 0.05, **Figure 3A**), Renal cortical protein expression of CYP2C11, another epoxygenase expressed in rat kidney, did not differ between the groups, but tended to be higher in clofibrate- compared with vehicle-treated animals (**Figure 3B**). The renal cortical protein expression of CYP4A, the w-hydroxylase that oxidizes arachidonic acid to 20-HETE, was approximately twofold higher in clofibrate- than vehicle-treated SHRSP (*P* < 0.01, **Figure 3C**).

### EFFECT OF CLOFIBRATE TREATMENT ON URINARY EXCRETION OF EETs AND DHETs

**FIGURE 4:**
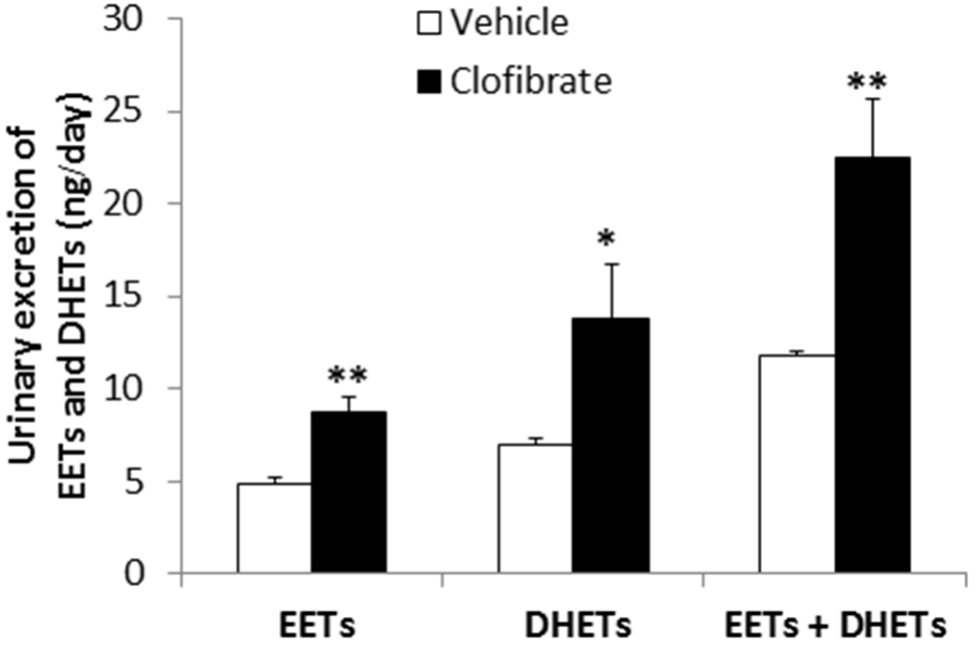
**Urinary excretion of EETs and DHETs in saline-drinking SHRSP treated with vehicle- (0.5% methylcellulose, *n* = 5) or clofibrate (200 mg/kg/day, p.o., *n* = 7) for 3 weeks.** Urinary excretion of EETs and/or DHETs was significantly higher in clofibrate-treated SHRSP. The sum of EETs and DHETs, an index of epoxygenase function, was also significantly increased with clofibrate treatment. Data were log-transformed to stabilize the variance before analyzing by Student’s *t*-test. The actual values are displayed and data are expressed as means ± SEM; *P < 0.01, **P < 0.005 vs. vehicle.

Urinary excretion of EETs was approximately twofold higher in clofibrate- compared with vehicle-treated SHRSP (*P* < 0.005, **Figure 4**), although the level of 14,15-EET was unaffected with clofibrate treatment (**Table 1**). Urinary excretion of DHETs was also significantly higher in clofibrate- compared with vehicle-treated SHRSP (*P* < 0.01, **Figure 4**) as were the levels of 11, 12-DHET and 14, 15-DHET. Total urinary excretion of EETs + DHETs, an index of renal epoxygenase function, was higher in the clofibrate-treated group (22.5 ± 3.2 ng/day) than the vehicle-treated group (11.8 ± 0.3 ng/day, *P* < 0.02). Urinary 20-HETE excretion was under the detection limit (10 pg/ml) of our LC/MS/MS.

**Table 1:** Effect of clofibrate (200 mg/kg/day, p.o.) on urinary excretion of EETs and DHETs (ng/day) in saline-drinking SHRSP.

	14,15-EET	11,12-EET	8,9-EET	5,6-EET
**Vehicle**	1.64 ± 0.15	1.26 ± 0.05	0.90 ± 0.13	1.03 ± 0.20
**Clofibrate**	2.64 ± 0.46	2.31 ± 0.26**	1.47 ± 0.16*	2.26 ± 0.33*

	**14,15-DHET**	**11,12-DHET**	**8,9-DHET**	**5,6-DHET**

**Vehicle**	3.34 ± 0.27	0.40 ± 0.04	0.62 ± 0.09	2.60 ± 0.41
**Clofibrate**	7.37 ± 1.52*	0.76 ± 0.12*	0.98 ± 0.48	4.68 ± 1.03

Data were log-transformed to stabilize the variance before analyzing by Student’s *t*-test. The actual values are displayed and data are expressed as means ± SEM; *n* = 5 in vehicle group and *n* = 7 in clofibrate group; *P < 0.05, **P < 0.002 vs. vehicle.

### EFFECT OF MS-PPOH TREATMENT ON SBP

The effects of *in vivo* MS-PPOH treatment on BP and kidney damage were examined in saline-drinking SHRSP. Animals were treated with MS-PPOH intravenously at a dosage of 20 mg/kg/day (Huang et al., 2006; Liclican et al., 2009). MS-PPOH treatment had negligible effects on SBP in saline-drinking SHRSP after 1 week, 160 ± 7 vs. 167 ± 6 mmHg, or 2 weeks of treatment, 171 ± 7 vs. 175 ± 8 mmHg, for vehicle vs. MS-PPOH, respectively.

### EFFECT OF MS-PPOH TREATMENT ON UPE AND RENAL HISTOLOGY

**FIGURE 5:**
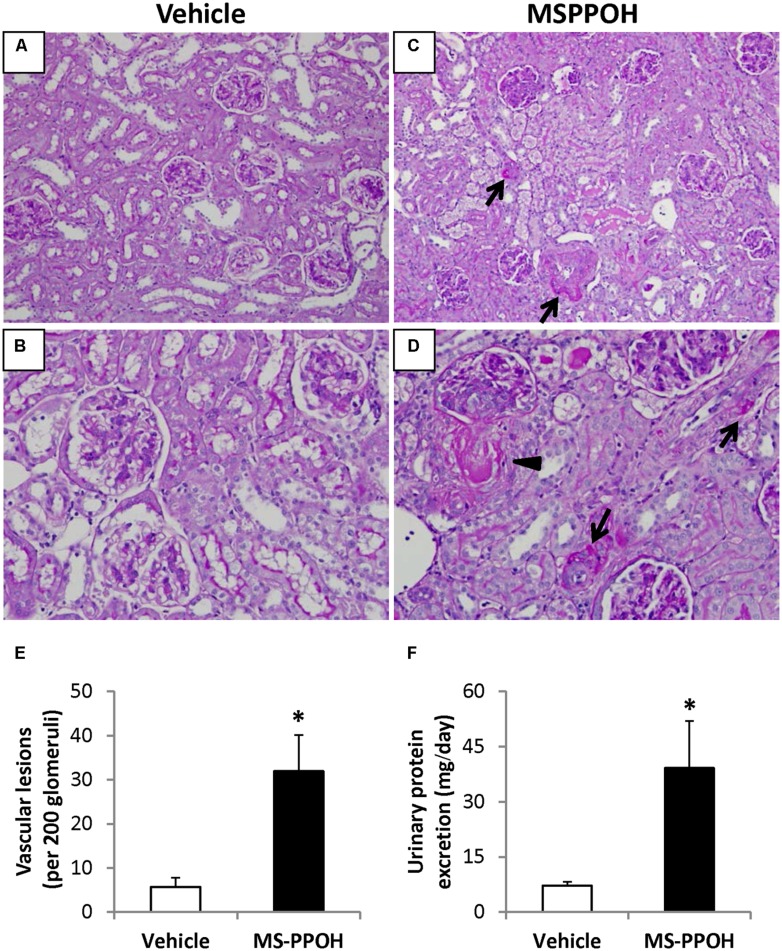
**(A–D)** Representative PAS-stained photomicrographs of renal cortex from two saline-drinking SHRSP treated with vehicle (45% hydroxypropyl β-cyclodextrin, *n* = 5; **A,B**) and two saline-drinking SHRSP treated with MS-PPOH (20 mg/kg/day, i.v., *n* = 7; **C,D**) for 2 weeks. Kidney sections from vehicle-treated SHRSP demonstrated very scattered and mild early microvascular lesions whereas kidney sections from MS-PPOH-treated SHRSP exhibited widespread and well-established lesions of malignant nephrosclerosis characterized by segmental to circumferential fibrinoid necrosis of microvessels (arrows). Arrow head points to a proliferative microvascular lesion; the adjacent glomerulus also shows segmental necrotizing and proliferative lesions from a MS-PPOH-treated SHRSP. These animals also exhibited focal but significant protein casts which were commensurate with proteinuria. Protein casts were generally absent in vehicle-treated animals. Magnification: ×20 **(A,C)** or ×40 **(B,D)**. **(E)** Quantification of renal microvessels exhibiting fibrinoid degeneration and/or proliferative lesions per field of 200 glomeruli. **(F)** Urinary protein excretion of SHRSP treated with vehicle or MS-PPOH for 2 weeks. Data are expressed as means ± SEM; *P < 0.05 vs. vehicle.

SBP was not altered with MS-PPOH treatment; however, four out of seven (4/7) MS-PPOH-treated SHRSP developed proteinuria (defined as UPE ≥ 20 mg/day). As expected, due to the young age and short period of HS intake, none (0/5) of the vehicle-treated SHRSP showed proteinuria. Overall UPE was higher in MS-PPOH-treated SHRSP compared with vehicle-treated animals (*P* < 0.01, **Figure 5F**). **Figures 5A,B** shows representative photomicrographs of PAS-stained kidney sections from vehicle-treated SHRSP, which demonstrated only scattered and mild renal vascular changes. In contrast, kidneys from MS-PPOH-treated SHRSP exhibited widespread lesions of malignant nephrosclerosis characterized by segmental to circumferential mural fibrinoid necrosis of microvessels with proliferative arteriopathy, which typically is seen in much older SHRSP with prolonged HS intake (**Figures 5C,D**). Proliferative lesions, which signify more severe microvascular damage, were present in five out of seven (5/7) kidneys from MS-PPOH-treated SHRSP, whereas none (0/5) of the vehicle-treated SHRSP developed such lesions (*P* < 0.05). The number of vessels exhibiting fibrinoid necrosis and/or proliferative lesions (**Figure 5E**) was markedly increased in kidneys from MS-PPOH-treated SHRSP compared with vehicle-treated SHRSP (*P* < 0.05). In addition to vascular damage, four out of seven (4/7) kidneys from MS-PPOH-treated SHRSP exhibited glomerular necrotizing and proliferative lesions, whereas none (0/5) of the vehicle-treated SHRSP developed glomerular lesions. Commensurate with UPE, the number of tubules presenting protein casts was markedly increased in kidneys from MS-PPOH-treated SHRSP (11 ± 3 per field of 200 glomeruli) compared with vehicle (2 ± 1 per field of 200 glomeruli, *P* < 0.05).

### EFFECT OF MS-PPOH TREATMENT ON URINARY EXCRETION OF EETs AND DHETs

**FIGURE 6:**
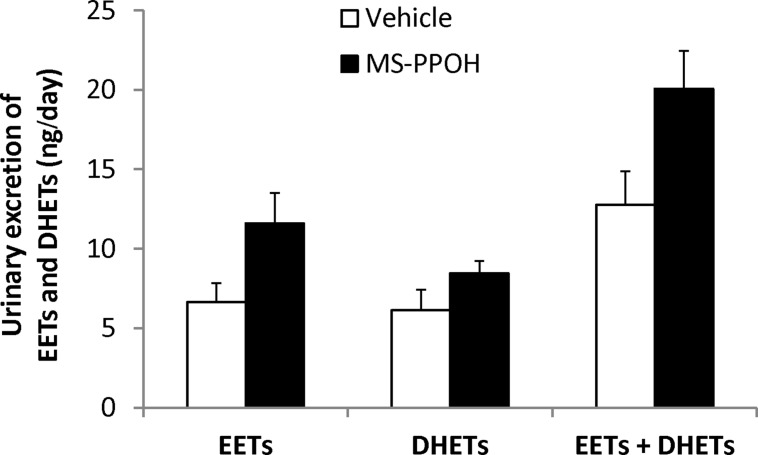
**Urinary excretion of EETs and DHETs in vehicle- (45% hydroxypropyl β-cyclodextrin, *n* = 5) and MS-PPOH- (20 mg/kg/day, i.v., *n* = 7) treated SHRSP at 2 weeks of treatment.** Urinary EET excretion and the sum of EETs + DHETs tended to be higher in MS-PPOH treated SHRSP, but these did not reach statistical significance. Data were log-transformed to stabilize the variance before analyzing by Student’s *t*-test. The actual values are displayed and data are expressed as means ± SEM.

Compared with vehicle-treated SHRSP, urinary excretion of EETs and DHETS was not different in MS-PPOH-treated SHRSP at 2 weeks of treatment (**Figure 6**). Total urinary excretion of EETs + DHETs tended to be higher in the MS-PPOH- (12.8 ± 2.1 ng/day) compared with vehicle-treated group (20.1 ± 2.4 ng/day). However, this also did not reach statistical significance (*P* = 0.052). None of the urinary levels of individual EET and DHET regioisomers were significantly altered with MS-PPOH treatment (**Table 2**).

**Table 2:** Effect of MS-PPOH (20 mg/kg/day, i.v.) on urinary excretion of EETs and DHETs (ng/day) in saline-drinking SHRSP.

	14,15-EET	11,12-EET	8,9-EET	5,6-EET
**Control**	2.50 ± 0.52	1.79 ± 0.26	1.42 ± 0.19	0.93 ± 0.25
**MS-PPOH**	4.71 ± 0.76	3.05 ± 0.51	2.78 ± 0.54	1.07 ± 0.24

	**14,15-DHET**	**11,12-DHET**	**8,9-DHET**	**5,6-DHET**

**Control**	2.62 ± 0.66	0.55 ± 0.10	0.48 ± 0.07	2.47 ± 0.51
**MS-PPOH**	3.34 ± 0.51	1.10 ± 0.22	0.69 ± 0.11	3.34 ± 0.34

*Data were log-transformed to stabilize the variance before analyzing by Student’s *t*-test. The actual values are displayed and data are expressed as means ± SEM; *n* = 5 in control group and *n* = 7 in MS-PPOH group.*

## DISCUSSION

In the present study, we used clofibrate as a pharmacological approach to elevate EET levels in SHRSP. In agreement with our previous study using a sEH inhibitor in young saline-drinking SHRSP (Li et al., 2008), treatment of clofibrate significantly reduced SBP and produced a renal protective effect. Fibrates have been reported to lower BP in several salt-loaded genetic models of hypertension (Roman et al., 1993; Shatara et al., 2000; Zhou et al., 2008), but have no effect on BP in normotensive Sprague–Dawley rats (Shatara et al., 2000). More importantly, clofibrate prevented DOCA-salt induced increases in BP in mice, indicating that the BP lowering effect of clofibrate is not necessarily secondary to a suppression of endogenous mineralocorticoid levels (Zhou et al., 2008). One of the underling mechanisms for the antihypertensive actions of fibrates has been suggested to be due to increased synthesis of 20-HETE (Alonso-Galicia et al., 1998; Zhou et al., 2008). However, fibric acid derivatives have also been reported to increase epoxygenase activity and EET production. Fenofibrate reduced BP and increased epoxygenase expression in double transgenic rats overexpressing both the human renin and angiotensinogen genes (Muller et al., 2004), as well as in Sprague–Dawley rats fed a high-fat diet (Huang et al., 2007) and Zucker diabetic fatty rats (Zhao et al., 2006; Zhao and Li, 2008). Fenofibrate treatment prevented brain and renal damage and reduced inflammation and oxidative stress of SHRSP (Gelosa et al., 2010). In the present study, renal cortical CYP2C23 protein expression and urinary excretion of EETs and DHETs were significantly increased by treatment with clofibrate. Clofibrate has also been reported to upregulate expression of cerebral CYP2C11 of SHRSP; however, CYP2C23 protein was not detected (Ying et al., 2008). These observations, taken together with our previous finding that sEH inhibition for two weeks prevented HS-induced increases in SBP in young SHRSP, suggest that EETs may contribute importantly to the BP lowering effect of clofibrate in saline-drinking SHRSP.

SHRSP develop proteinuria, glomerular damage, and renal microvascular lesions characteristic of thrombotic microangiopathy in malignant nephrosclerosis with age. These renal pathologic changes are markedly accelerated by salt-loading and standard rodent diet when started at approximately 7 weeks of age. SHRSP manifest severe renal damage after 4 weeks on HS intake and die of strokes, primarily hemorrhagic infarcts, by 13–15 weeks of age (6–8 weeks of HS intake; Stier et al., 1991, 1993). In the present study, 3 weeks of HS intake produced mild to moderated vascular fibrinoid degeneration in vehicle-treated SHRSP which was consistent with the progression to severe renal damage (almost 15% of glomeruli and 20 vascular lesions per field of 100 glomeruli) after 4 weeks of HS intake in SHRSP (Zuckerman et al., 1997). Clofibrate treatment reduced UPE and markedly diminished renal glomerular and vascular damage. These results are consistent with our finding that sEH inhibition ameliorated early salt-sensitive renal damage in saline-drinking SHRSP, which supports a protective role for EETs against salt-sensitive renal damage (Li et al., 2008). Fibrates have been reported to ameliorate renal damage in other animal models. In double transgenic rats overexpressing both human renin and angiotensinogen genes, fenofibrate reduced renal collagen IV expression and leukocyte infiltration (Muller et al., 2004). Fenofibrate also attenuated glomerular hypertrophy and collagen accumulation in Zucker diabetic fatty rats (Zhao and Li, 2008). Treatment with fenofibrate has been reported to reduce the number of abnormal glomeruli and to diminish the degree of mesangial expansion and glomerulosclerosis in Dahl salt-sensitive rats (Wilson et al., 1998). It is well known that EETs possess anti-inflammatory and anti-proliferative activity in vascular smooth muscle (McGiff and Ferreri, 2007). Recently, 8,9-EET has been shown to prevent the focal segmental glomerulosclerosis-induced increase in glomerular albumin permeability *in vitro* (Sharma et al., 2009). Thus, we reasoned that the reduction of UPE and renal damage by clofibrate may be related to the increase in levels of EETs. To be noted, in the present study, since BP was appreciably lowered by clofibrate treatment, we cannot separate direct vasculoprotective effects of increasing EET levels from BP lowering effects on the development of renal damage in these rats. Clofibrate treatment was, however, reported to increase cerebral blood flow, prevent stroke and prolong survival of SHRSP (Ying et al., 2008).

Although we found an increase in CYP4A protein expression, unlike urinary EETs, 20-HETE was undetectable in the urine in either vehicle- or clofibrate-treated SHRSP as measured by LC/MS/MS. Like 20-HETE, EETs possess diuretic and natriuretic properties (Houillier et al., 1996; Wei et al., 2004). However, unlike 20-HETE which can cause vasoconstriction (McGiff and Ferreri, 2007), EETs produce vasodilatation of renal arterioles and may serve as the endothelium-derived hyperpolarizing factor in the vasculature (Eckman et al., 1998; Huang et al., 2005). 20-HETE has also been reported to play a role in controlling the glomerular permeability barrier to albumin, which mimics the effect of 8,9-EET at the glomerular permeability barrier (McCarthy et al., 2005). A recent study demonstrated that introgression of the CYP4A genes from Lewis rats into the Dahl salt-sensitive rats increased renal formation of 20-HETE and attenuated the development of hypertension and renal disease (Williams et al., 2008). In the present study, although urinary 20-HETE was undetectable, renal cortical CYP4A protein expression was induced by clofibrate treatment in saline-drinking SHRSP. Thus, we cannot rule out a possible contribution of CYP4A/20-HETE to the renal protective effects of clofibrate. Indeed, it may be the combined effects of EETs and 20-HETE which is responsible for the marked renal protection in saline-drinking SHRSP as both EETs and 20-HETE promote sodium excretion and act to limit glomerular proteinuria.

We also examined inhibition of epoxygenase to determine if loss of EETs would promote end-organ damage in saline-drinking SHRSP. MS-PPOH has been identified as a potent and selective inhibitor of epoxygenases *in vitro* and* in vivo* (Brand-Schieber et al., 2000). Rat renal microsomal epoxygenase activity was inhibited for up to 6 h after a single i.v. bolus injection of MS-PPOH (5 mg; Brand-Schieber et al., 2000) and we have previously shown that treatment with MS-PPOH (20 mg/kg/day, i.v.) for 6 days significantly reduced renal levels of EETs in Dahl salt-resistant rats on 2% NaCl drinking solution (Liclican et al., 2009). Consistent with this hypothesis, we found that *in vivo* epoxygenase inhibition with MS-PPOH in young SHRSP increased UPE and accelerated the development of renal damage consistent with malignant nephrosclerosis, as typically seen in older SHRSP after at least 4 weeks of salt-loading (Rocha et al., 1998). Vehicle treatment was associated with only mild renal damage as we have seen previously with 2 weeks of HS intake (Zuckerman et al., 1997; Li et al., 2008). Moreover, the microvascular damage with MS-PPOH treatment was significantly greater than that present in the vehicle-treated SHRSP in the clofibrate study, despite the fact that these animals were younger (9 vs. 10 weeks of age) and received HS intake for a shorter period of time than in the clofibrate study (11 days vs. 3 weeks). The data therefore suggest that EETs play a protective role against the development of salt-sensitive renal damage in saline-drinking SHRSP.

It has been previously reported that *in vivo* MS-PPOH treatment significantly increased BP, renal vascular resistance and sodium balance in pregnant rats (Huang et al., 2006) and increased BP in Dahl salt-resistant rats on HS intake (Liclican et al., 2009). In the present study, however, SBP was not further elevated with MS-PPOH treatment. This discrepancy may reflect a strain difference or a difference in pre-treatment BP, since the earlier two animal models were normotensive whereas SHRSP were hypertensive before the MS-PPOH treatment. Although SBP was not increased with MS-PPOH treatment in the present study, our findings are consistent with the notion that endogenous EETs may serve a renal vascular protective role independent of an effect on BP (Olearczyk et al., 2009).

Urinary excretion of EETs and DHETs was not reduced with MS-PPOH treatment in the present study. However, the appearance of heavy proteinuria in MS-PPOH-treated SHRSP may have masked this affect. The majority of plasma EETs bind to plasma albumin non-covalently in the form of fatty acid–albumin complex (Saifer and Goldman, 1961). Therefore, the EETs that bind to urinary albumin can be extracted by our method without the requirement for alkaline hydrolysis. Since some MS-PPOH-treated animals developed moderate to heavy proteinuria, EETs that bind with urinary albumin may have masked the reduction of EETs by epoxygenase inhibition with MS-PPOH. In addition to inhibition of epoxygenases, MS-PPOH has been reported to compete for the binding of a radiolabeled EET antagonist to cell membranes and inhibit 14,15-EET-induced relaxation of bovine coronary artery, suggesting a secondary mechanism by which MS-PPOH has an inhibitory action on the epoxygenase-EET pathway (Chen et al., 2009).

In conclusion, clofibrate treatment reduced SBP, UPE, and renal damage in saline-drinking SHRSP, effects that may be related to increased levels of EETs as reflected by up-regulation of renal cortical epoxygenase expression and increased urinary excretion of EETs. In contrast, the CYP450 epoxygenase inhibitor, MS-PPOH, increased UPE and accelerated the development of renal damage in saline-drinking SHRSP without altering urinary EET levels. Together with our previous finding that sEH inhibition prevented early microvascular damage in saline-drinking SHRSP (Li et al., 2008), these results suggest that pharmacological manipulation of epoxygenase results in divergent effects on renal damage such that inhibition promotes injury and increasing EET synthesis reduces injury. Interventions to increase renal EET levels may provide therapeutic strategies for treating salt-sensitive hypertension and renal damage.

## Conflict of Interest Statement

The authors declare that the research was conducted in the absence of any commercial or financial relationships that could be construed as a potential conflict of interest.

## ABBREVIATIONS

AUDA: 2-(3-adamantan-1-yl-ureido)-dodecanoic acid
CYP: cytochrome P450
DHETs: dihydroxyeicosatrienoic acids
EETs: epoxyeicosatrienoic acids
ENaC: epithelial sodium channel
20-HETE: 20-hydroxyeicosatetraenoic acid
HS: high-salt
MS-PPOH: *N*-methylsulfonyl-6-(2-propargyloxyphenyl)hexanamide
PAS: periodic acid-Schiff reagent
SBP: systolic blood pressure
sEH: soluble epoxide hydrolase
SHRSP: stroke-prone spontaneously hypertensive rat
SPRD: stroke-prone rodent diet
UPE: urinary protein excretion.
